# Relationship of the Acoustic Startle Response and Its Modulation to Adaptive and Maladaptive Behaviors in Typically Developing Children and Those With Autism Spectrum Disorders: A Pilot Study

**DOI:** 10.3389/fnhum.2019.00005

**Published:** 2019-01-22

**Authors:** Ken Ebishima, Hidetoshi Takahashi, Andrew Stickley, Takayuki Nakahachi, Tomiki Sumiyoshi, Yoko Kamio

**Affiliations:** ^1^National Center of Neurology and Psychiatry, Department of Preventive Intervention for Psychiatric Disorders, National Institute of Mental Health, Tokyo, Japan; ^2^Integrative Brain Imaging Center, National Center of Neurology and Psychiatry, Department of Advanced Neuroimaging, Tokyo, Japan; ^3^Stockholm Center for Health and Social Change (SCOHOST), Södertörn University, Huddinge, Sweden

**Keywords:** acoustic startle reflex, adaptive behavior, hypersensitivity, autism spectrum disorder, neurophysiology, sensorimotor gating

## Abstract

**Background**: Autism spectrum disorder (ASD) is associated with persistent impairments in adaptive functioning across multiple domains of daily life. Thus, investigation of the biological background of both adaptive and maladaptive behaviors may shed light on developing effective interventions for improving social adaptation in ASD. In this study, we examined the relationship between adaptive/maladaptive behaviors and the acoustic startle response (ASR) and its modulation, which are promising neurophysiological markers for ASD translational research.

**Method**: We investigated the ASR and its modulation in 11 children with ASD and 18 with typical development (TD), analyzing the relationship between startle measures and adaptive/maladaptive behaviors assessed with the Vineland Adaptive Behavior Scales (VABS) Second Edition.

**Results**: Peak-ASR latency was negatively correlated with the VABS total score and socialization domain score of adaptive behaviors, while the ASR magnitude for relatively weak stimuli of 75–85 dB was positively correlated with VABS maladaptive behavior scores. Prepulse inhibition (PPI) at the prepulse intensity of 70–75 dB was also correlated with VABS maladaptive behavior. However, these relationships did not remain significant after adjustment for multiple comparisons.

**Conclusions**: Our results indicate that the prolonged peak-ASR latency of ASD children might be associated with impairment in the developmental level of adaptive behavior, and that the greater ASR magnitude to relatively weak acoustic stimuli and smaller PPI of ASD children might increase the risk of maladaptive behavior. Future studies that have larger sample sizes will be important for further elucidating the neurophysiological factors that underpin adaptive as well as maladaptive behaviors in ASD.

## Introduction

Autism spectrum disorder (ASD) is associated with persistent impairments in adaptive functioning across multiple domains including social, communicative, occupational, or other important areas of daily life (American Psychiatric Association, 2000). Development of adaptive behaviors and prevention of maladaptive behavior are primary and fundamental intervention aims in ASD.

Sensory abnormalities have often been reported as symptoms of ASD (Lai et al., 2014), and also reported to be related to adaptive/maladaptive behaviors (Lane et al., 2010; Schauder and Bennetto, 2016). Thus, investigating the biological background of sensory abnormalities and its relationship to adaptive as well as maladaptive ASD behaviors might uncover the neurobiological cascade of adaptive/maladaptive behaviors, which may shed light on developing effective interventions for improving social adaptation in ASD, however, there is a dearth of research on this issue.

Recently, we reported that the acoustic startle reflex (ASR) and its modulation, such as sensorimotor gating evaluated as prepulse inhibition (PPI), might serve as a promising and quantitative neurophysiological endophenotype of sensory processing and act as a diagnostic marker of ASD as well as comorbid psychiatric conditions (Takahashi et al., 2014, 2016). A prolonged peak-ASR latency (Takahashi et al., 2014, 2016) and a greater ASR magnitude (Takahashi et al., 2014, 2016) in response to weak stimuli of 65–85 dB was found in children with ASD compared to those with typical development (TD), and these indices were related to autistic traits and emotional/behavioral difficulties in ASD children (Takahashi et al., 2016).

Building on this earlier research, in this study, we investigated the influence of the ASR and its modulation including PPI on adaptive/maladaptive behaviors, assessed with the Japanese version (Kuroda et al., 2014) of the Vineland Adaptive Behavior Scales (VABS), in children with ASD and those with TD, in order to examine the neurophysiological background of these behaviors in ASD. We hypothesized that adaptive and maladaptive behaviors might be related to different aspects of the ASR and its modulation. We investigated several ASR intensities, as a greater ASR to relatively weak stimuli has been related to several clinical features in children with ASD (Takahashi et al., 2016).

## Materials and Methods

### Participants

Eleven Japanese children with ASD (age 8–16 years old; eight boys) and 18 typically developing (TD) Japanese children (age 8–16 years old; 12 boys) participated in the study. Experienced child psychiatrists assigned diagnoses after reviewing medical records and performing clinical interviews based on the Diagnostic and Statistical Manual of Mental Disorders, Fourth Edition, Text Revision (American Psychiatric Association, 2000). Diagnoses were confirmed using the Autism Diagnostic Interview-Revised (Lord et al., 1995) and the Autism Diagnostic Observation Schedule (Lord et al., 2000). Intelligence quotient (IQ) was assessed with the Wechsler Intelligence Scale for Children-Third Revision (WISC-III: Wechsler, 1991). Neither sex (χ^2^ = 0.117, *df* = 1, *p* = 0.732), age (age in years; ASD 11.6 ± 2.1; TD 11.4 ± 2.1; *U* = 91, *p* = 0.719), or estimated IQ (ASD 85.6 ± 40.1; TD 95.6 ± 32.5; *U* = 44.5, *p* = 0.317) differed significantly between the two groups. Additionally, results from the WISC-III (Wechsler, 1991) also showed that the estimated IQ for every child in the study was above 70. No child smoked and none were currently being medicated with psychotropic substances. Children were excluded from the study if they had any degree of hearing loss or abnormalities of the central nervous system apart from autism. Additionally, members of the TD group were excluded if they had previous or current psychiatric diagnoses or a learning disability.

### Ethics Approval and Consent to Participate

The Research Ethics Committee of the National Center of Neurology and Psychiatry, Japan granted institutional review-board approval for the study (#A2013-112) and it was undertaken in accordance with the principles laid out in the Helsinki Declaration 1964 and its subsequent amendments. The study procedures were fully explained to all participants and their parents who then provided written informed consent before being included in the study.

### Startle Response Measurement

A commercial computerized human startle-response monitoring system (Startle Eyeblink Reflex Analysis System Map1155SYS, Nihonsanteku Co., Osaka, Japan) was used to deliver acoustic startle stimuli and to record and score the corresponding electromyographic activity. The specific methods for stimulus presentation and eyeblink acquisition have been described in detail previously (Takahashi et al., 2017, 2018). The following startle measures were examined: (1) ASR65, ASR75, ASR85, ASR95, and ASR105: average ASR eyeblink magnitude in response to pulse intensities of 65, 75, 85, 95, and 105 dB SPL, respectively; (2) the peak-ASR latency, defined as the average peak-ASR latency across trials with an ASR larger than 60 μV; (3) habituation of the ASR during the session, defined as the percentage of ASR amplitude reduction at 105 dB SPL and (4) PPI65, PPI70, PPI75: PPI at prepulse intensities of 65, 70, and 75 dB SPL, respectively. The PPI at each prepulse intensity was defined as the percentage of amplitude reduction between pulse alone and pulse with prepulse trials. Trials were discarded if the voltage of their peak electromyographic activity was above 60 μV within a latency window of 0–20 ms following the startle-eliciting stimulus onset. Startle measures were not calculated for conditions in which more than half of the trials had been discarded.

### Assessment of Adaptive and Maladaptive Behaviors

The children’s adaptive and maladaptive behaviors were assessed with the Japanese version (Kuroda et al., 2014) of the VABS Second Edition (Sparrow et al., 2005), which was administered to the mothers of the participants by a child psychiatrist. The VABS Second Edition is composed of two parts, an “adaptive behavior evaluation” part, which measures the level of adaptive behavior [the skills needed by individuals to function and be self-sufficient within their everyday environments (Sparrow et al., 2005)], and a “maladaptive behavior evaluation” part, which measures behavior that is problematic with respect to individual social life. Among the four different domains of adaptive behavior evaluation, communication (conceptual), socialization (social), and daily living (practical) adaptive skills were evaluated, whereas the motor adaptive skills domain was not used in this study as this score is designed to be evaluated only in participants who are aged 6 years or under, or over 50 years. Each of these domains comprises subdomains (receptive, expressive, and written skills in communication; personal, domestic, and community skills of daily living; interpersonal relationships, play and leisure time, and coping skills of socialization; and gross and fine motor skills) with item sets assessing specific content areas (e.g., adaptive skills). Maladaptive behavior evaluation comprises three domains (internalizing problems, externalizing problems, and others). We obtained total standard scores (*M* = 100, *SD* = 15) as well as scores for each of the adaptive behavior domains, and a v-scale score (*M* = 15, *SD* = 3) for maladaptive behavior. The higher the adaptive behavior standard score, the higher the adaptive behavior level, the higher the maladaptive behavior v-scale score, the higher the risk of maladaptation in life.

### Statistical Analysis

Chi-square tests were used to examine categorical proportions. As most of the variables relating to the ASR and VABS scores were not normally distributed, nonparametric analyses were performed. The Mann-Whitney *U* test was used to compare the median scores of parameter values. Spearman’s rank order correlation coefficients were used to examine the relationships between variables. SPSS Ver. 22 (IBM Japan, Tokyo, Japan) was used to perform all statistical analyses with the level of statistical significance set at *p* < 0.05. A Bonferroni correction was subsequently used to adjust significance levels for multiple comparisons.

## Results

### Differences in Adaptive/Maladaptive Behaviors and Startle Measures Between Children With Autism Spectrum Disorders and Controls

The VABS scores and ASR measures for both groups are presented in Table 1. All VABS scores except the maladaptive behavior score, were significantly lower in the ASD group than in the controls. Children with ASD had a significantly prolonged peak-ASR latency. Additionally, their ASR75 was significantly greater. A trend towards greater ASR65 and ASR85 was also observed in ASD children. There were no statistically significant differences observed between the groups for other ASR measures, including ASR modulation of habituation and PPI at all prepulse intensities. However, after correction for multiple comparisons, only group differences in the VABS total and socialization domain scores remained significant.

**Table 1:** Adaptive/maladaptive behavior scores and startle measures.

	Typical development (*N* = 18)		Autism spectrum disorders (*N* = 11)				
	Median	Inter-quartile range	Median	Inter-quartile range	*U*	*p*-value	Effect size (*r*)
Vineland Adaptive Behavior Scales Adaptive behavior							
Total	104	92.5–128.0	91	69.0–94.0	17.0	<0.001	0.69
Communication	103	77.0–112.5	75	66.0–88.0	41.0	0.009	0.49
Socialization	99	91.5–119.5	83	79.0–92.0	8.0	<0.001	0.76
Daily living	110	104.0–132.0	98	72.0–117.0	41.0	0.009	0.49
Maladaptive behavior	16	13.5–17.5	18	13.0–20.0	75.5	0.287	0.20
Peak startle latency (ms)	72.4	67.0–79.5	85.0	71.9–90.1	36.0	0.005	0.53
Acoustic startle magnitude (μV)							
65 dB	30.4	20.1–35.9	37.6	35.6–46.3	61.5	0.092	0.31
75 dB	28.5	18.9–40.2	46.0	40.1–56.4	52.0	0.035	0.39
85 dB	37.7	32.4–44.3	57.1	50.0–81.5	57.0	0.059	0.35
95 dB	47.5	28.9–65.1	47.3	44.0–56.3	96.0	0.893	0.03
105 dB	75.9	61.0–82.0	65.7	51.0–149.6	84.0	0.500	0.13
Habituation (%)^†^	28.9	13.0–36.3	22.8	2.7–31.0	52.0	1.000	0.00
Prepulse inhibition (%)^†^							
65 dB	20.4	4.3–37.7	14.2	5.2–23.1	66.0	0.618	0.10
70 dB	30.2	13.3–42.4	39.8	5.5–48.4	87.5	0.778	0.05
75 dB	42.3	29.7–48.8	49.2	16.8–59.6	89.5	0.851	0.04

*Mann-Whitney U test. The level of statistical significance was set at p < 0.003 after a Bonferroni correction. ^†^Number of participants (typical development, TD: autism spectrum disorders, ASDs) for habituation = 13: 8; Prepulse *inhibition* (65-dB prepulse) = 15: 10; (70-dB prepulse) = 17: 11; (75-dB prepulse) = 17: 11*.

### Relationship of Adaptive/Maladaptive Behaviors to Startle Measures

**Figure 1:**
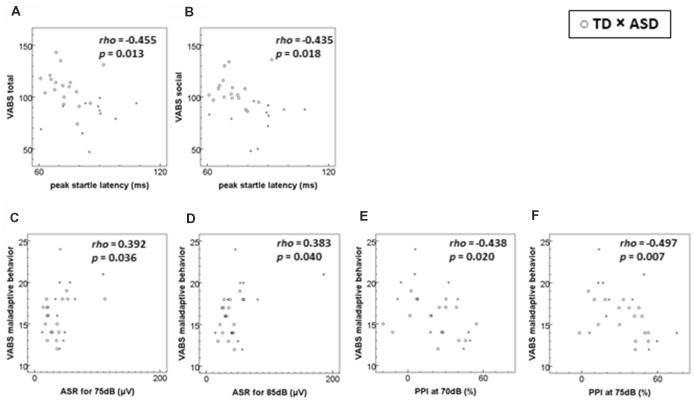
Scatterplots of significant relationships of adaptive/maladaptive behavior scores to startle measures. **(A)** VABS total score to peak startle latency. **(B)** VABS socialization (social) domain score to peak startle latency. **(C)** VABS maladaptive behavior score to ASR for 75 dB stimuli. **(D)** VABS maladaptive behavior score to ASR for 85 dB stimuli. **(E)** VABS maladaptive behavior score to PPI at 70 dB prepulse. **(F)** VABS maladaptive behavior score to PPI at 75 dB prepulse. ASD, autism spectrum disorder; ASR, acoustic startle response magnitude; PPI, prepulse inhibition; TD, typical development; VABS, Vineland Adaptive Behavior Scales. The rho and the *p*-values are derived from Spearman’s rank order correlations. The level of statistical significance was set at *p* < 0.001 after a Bonferroni correction. Number of participants (TD:ASD): **(A–D)** 18:11; **(E,F)** 17:11.

Figure 1 shows the scatter plot of significant relationships between the VABS scores and ASR measures for all subjects. The peak-ASR latency was negatively correlated with the VABS total score and socialization domain score, while the ASR magnitudes for 75–85 dB stimulation were positively correlated with the VABS maladaptive behavior score. PPI at the prepulse intensity of 70–75 dB was also correlated negatively with the VABS maladaptive behavior score. However, these relationships did not remain significant after adjustment for multiple comparisons.

In the ASD group, we found a significant correlation between the VABS maladaptive behavior score and PPI70 (ρ = −0.651, *p* = 0.030). This relationship did not remain significant after adjustment for multiple comparisons. No other significant relationships between the ASR measures and VABS scores were observed in either group.

## Discussion

In this pilot study, we found possible relationships between adaptive and maladaptive behaviors and different aspects of the ASR in children with ASD and TD. Impaired adaptive behavior evaluated by the VABS total and socialization domain scores was negatively related to peak-ASR latency, while the VABS maladaptive behavior score was related not only to a greater startle magnitude to weak acoustic stimuli of 75 and 85 dB, but also to PPI.

Possible relationships were observed between impaired adaptive behaviors, especially for the socialization domain, and prolonged peak-ASR latency. Regarding the VABS adaptive behavior domains, previous research has highlighted that the socialization domain is consistently impaired in people with ASD irrespective of their cognitive level (Volkmar et al., 1987; Carter et al., 1998; Fenton et al., 2003; Klin et al., 2007; Perry et al., 2009). Thus, prolonged peak-ASR latency might serve as a possible marker of the impaired adaptive behavior which is seen in ASD.

We also found a possible relationship of maladaptive behavior to a greater startle response to relatively weak acoustic stimuli as well as PPI. The PPI is considered to be a stable neurophysiological marker, which continues to develop to full maturation until around 8 years of age (Takahashi et al., 2011). PPI impairment has been noted in several psychiatric diseases, such as schizophrenia, obsessive-compulsive disorder and posttraumatic stress disorder (Takahashi et al., 2011), however, consistent results have not been obtained with respect to the PPI of ASD in previous studies (Takahashi and Kamio, 2018), and we did not find a PPI difference between ASD and TD children. Thus, maladaptive behavior might be explained not only by greater hyper-reactivity to relatively weak stimuli, which was found in ASD, but also by impairment of sensorimotor gating, which is not specific to ASD. This suggests that interventions for maladaptive behavior might be better started as early as possible before full PPI development has occurred.

The small sample size was a major limitation of this study. As significant ASR 65 group differences that were reported in previous studies (Takahashi et al., 2014, 2016) were not observed in the current study, and most of the associations between the ASR measures and adaptive/maladaptive behaviors became non-significant when the children were divided into groups, the small sample size might have affected the results. Further, although gender differences exist in ASD (Lai et al., 2014), this study consisted mainly of boys, while the age span of the children was also rather large. In addition, adaptive behaviors were evaluated with standardized scores while maladaptive behaviors were assessed with v-scale scores, and these scoring differences might also have affected the results. Future studies that have larger sample sizes of both sexes where the age range is narrower and that use other assessment tools standardized for maladaptive behavior will be important for further elucidating the neurophysiological factors that underpin adaptive as well as maladaptive behaviors in ASD.

## Data Availability

The datasets generated for this study are available on request to the corresponding author.

## Author Contributions

HT, KE and YK conceived and designed the experiments and analyzed the data. HT and YK supervised the project and confirmed the diagnoses. KE, HT and TN performed the experiments. KE, HT, AS, TN, TS and YK wrote the manuscript. All authors read and approved the final manuscript.

## Conflict of Interest Statement

The authors declare that the research was conducted in the absence of any commercial or financial relationships that could be construed as a potential conflict of interest.
